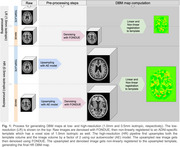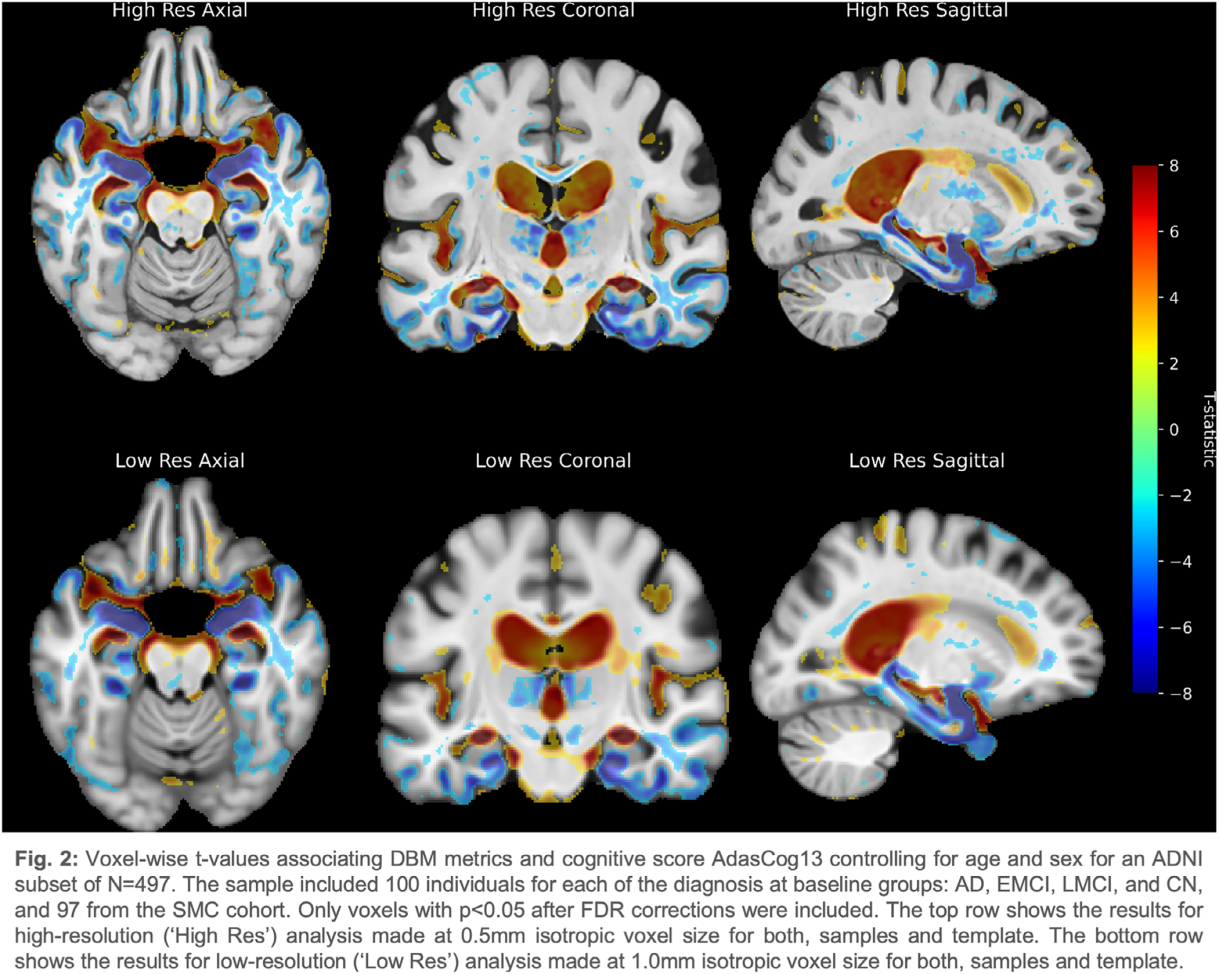# Enhancing voxel‐level morphometry through Deep Learning‐based MRI Super‐Resolution for detecting Alzheimer's Disease related atrophy

**DOI:** 10.1002/alz70856_107471

**Published:** 2026-01-09

**Authors:** Walter Adame‐Gonzalez, Roqaie Moqadam, Yashar Zeighami, Mahsa Dadar

**Affiliations:** ^1^ McGill University, Montreal, QC, Canada; ^2^ Douglas Mental Health University Institute, Montreal, QC, Canada; ^3^ University of Montreal, Montréal, QC, Canada; ^4^ Douglas Mental Health University Institute, Montréal, QC, Canada; ^5^ Department of Psychiatry, McGill University, Montréal, QC, Canada; ^6^ McGill University, Montréal, QC, Canada

## Abstract

**Background:**

Alzheimer's Disease (AD) is characterized by the accumulation of Amyloid‐beta plaques and hyperphosphorylated‐tau neuro‐fibrillary tangles (NFT). In early stages of the disease, grey matter loss and proteinopathy is localized to the entorhinal cortices, nucleus basalis of Meynert, and hippocampus (Shafiee et. al. 2024). Additionally, atrophy in these regions has been shown to mediate cognitive decline (Xia et. al. 2024). Deformation Based Morphometry (DBM) is a widely‐used technique for modelling voxel‐wise volume changes with respect to a common template using Magnetic Resonance Imaging (MRI) data. However, DBM is constrained by MRI voxel resolution, making the study of smaller structures more challenging as commonly used MRI images are acquired at ∼1 mm3 voxel size.

**Method:**

We used baseline T1‐weighted MRI images from an ADNI subsample of age‐, sex‐, and diagnosis balanced individuals (*N* = 497). We produced high‐resolution images by upsampling and denoising the 497 images and the 1 mm3 template using an in‐house deep learning method based on autoencoders. For both resolutions, voxel‐wise DBM maps were obtained following preprocessing, linear and non‐linear registration to an ADNI‐specific template (Figure 1). Similarly, voxel‐wise linear regressions were performed at both resolutions. We then computed voxel‐wise linear regression models to assess the relationship between ADASCog13 scores and DBM maps, including age and sex as covariates. The results were corrected for multiple comparisons using the false discovery rate (FDR) method.

**Result:**

While the significant regions were consistent between the two models, the high‐resolution models yielded more significant voxels (19.23% compared to 18.52%) after FDR correction. In addition, in the high‐resolution models, the atrophy patterns were better defined and more localized to the grey matter in temporal cortex, entorhinal cortices, and hippocampus regions, suggesting a reduction in partial volume effects, particularly in the grey‐to‐white matter interface. For example, the hippocampus head showed significant atrophy in the dentate gyrus and CA4 subfields with the 0.5 mm3 approach that was not visible at 1.0 mm3 resolution (Figure 2, coronal view).

**Conclusion:**

The proposed super resolution method can enhance our ability to detect subtle atrophy patterns that are most relevant at early stages of the disease.